# COPD: real-world evidence on single-inhaler triple therapy and the emergence of biologic therapy

**DOI:** 10.3389/fphar.2026.1983112

**Published:** 2026-10-23

**Authors:** Beatrice Da Re, Nicolò Reccardini, Giorgio Monteleone, Eugenio Capparelli, Michael Hughes, Giulia Bandini, Yukai Wang, Paola Confalonieri, Francesco Salton, Marco Confalonieri, Umberto Semenzato, Barbara Ruaro

**Affiliations:** 1 Department of Medical, Surgical and Health Sciences, University of Trieste, Trieste, Italy; 2 Department of Pulmonology, Santa Maria Degli Angeli Hospital, Pordenone, Italy; 3 Department of Pulmonary Rehabilitation, Azienda Sanitaria Universitaria Friuli Centrale, Udine, Italy; 4 Department of Pneumology, Ruhrlandklinik, University Hospital Essen, University of Duisburg-Essen, Essen, Germany; 5 Rheumatology Unit, ASST Ovest Milanese, Legnano Hospital, Milan, Italy; 6 Centre for Musculoskeletal Research, Division of Musculoskeletal and Dermatological Science, School of Biological Sciences, Faculty of Biological Medicine and Health, the University of Manchester, Manchester Academic Health Science Centre, Manchester, United Kingdom; 7 Department of Rheumatology, Northern Care Alliance NHS Foundation Trust, Salford Care Organisation, Salford, United Kingdom; 8 NIHR Manchester Biomedical Research Centre, Manchester University NHS Foundation Trust, Manchester, United Kingdom; 9 Department of Experimental and Clinical Medicine, Division of Internal Medicine, Azienda Ospedaliera Universitaria Careggi, University of Florence, Florence, Italy; 10 Department of Rheumatology and Immunology, Shantou Central Hospital, Shantou, China; 11 Department of Pulmonology, University Hospital of Cattinara, Trieste, Italy; 12 Department of Cardiac, Respiratory Medicine, Thoracic, Vascular Sciences and Public Health, University of Padova, Padua, Italy

**Keywords:** biological therapy, COPD, dupilumab, eosinophils, mepolizumab, real-world evidence, single-inhaler triple therapy (SITT), tozorakimab

## Abstract

Chronic obstructive pulmonary disease (COPD) remains a leading cause of morbidity and mortality worldwide. Despite major pharmacological advances, a substantial gap persists between guideline recommendations and routine clinical practice, particularly after exacerbations. This narrative review summarizes current evidence supporting personalized pharmacological escalation in COPD, focusing on single-inhaler triple therapy (SITT) and the emergence of biologic therapy. SITT, combining an inhaled corticosteroid, a long-acting β_2_-agonist and a long-acting muscarinic antagonist, is now the preferred inhaled strategy for patients requiring ICS-containing triple therapy. Real-world studies indicate that, compared with multiple-inhaler triple therapy, SITT is associated with better adherence and persistence, lower exacerbation rates and improved clinical outcomes, although observational findings require cautious causal interpretation. In parallel, COPD has entered the era of targeted biologic therapy. Dupilumab was the first biologic approved for COPD, following the BOREAS and NOTUS trials in patients with chronic bronchitis, blood eosinophils ≥300 cells/µL and persistent exacerbations despite triple therapy. Mepolizumab has more recently provided robust phase 3 evidence in eosinophilic COPD through the MATINEE trial. Early real-world dupilumab studies further suggest potential effectiveness in selected patients under-represented in pivotal trials, including those with advanced age, severe airflow limitation, oxygen dependence or high mucus burden. Beyond canonical type 2 pathways, IL-33 inhibition with tozorakimab may further broaden targeted treatment to COPD phenotypes without a clear eosinophilic signature. Integrating these advances within a treatable-traits framework may help optimize patient selection, close post-exacerbation therapeutic gaps, and align pharmacological escalation with comorbidity management, adherence, inhaler technique and rehabilitation needs.

## 1 Introduction

Chronic obstructive pulmonary disease (COPD) is a heterogeneous, progressive respiratory condition characterized by persistent airflow limitation and abnormal inflammatory responses to inhaled noxious particles or gases, most notably tobacco smoke (Global Initiative for Chronic Obstructive Lung Disease, 2025). It affects an estimated 300 million individuals worldwide and remains one of the leading causes of morbidity and mortality globally (Global Initiative for Chronic Obstructive Lung Disease, 2025; Adeloye et al., 2022). Exacerbations—episodes of acute worsening of respiratory symptoms—represent a major driver of disease progression, hospitalization, and mortality. Indeed, each moderate or severe exacerbation is associated with an accelerated decline in lung function, increased cardiovascular risk, and a measurable rise in short- and long-term mortality (Gladysheva et al., 2010; Soler-Cataluña et al., 2005).

For over 2 decades, the pharmacological cornerstone of COPD management has been inhaled bronchodilator therapy, progressively escalated according to symptom burden and exacerbation history. More recently, COPD treatment has increasingly moved from a stepwise escalation model towards a more personalized approach based on treatable traits, including exacerbation risk, blood eosinophil counts, chronic bronchitis, hyperinflation, inhaler adherence, and comorbidities. Within this evolving framework, two major pharmacological developments have reshaped the therapeutic landscape: the consolidation of single-inhaler triple therapy (SITT), combining an inhaled corticosteroid (ICS), a long-acting β_2_-agonist (LABA), and a long-acting muscarinic antagonist (LAMA), as the preferred approach for patients requiring triple therapy; and the emergence of biologic therapies targeting type 2/eosinophilic inflammation as add-on options for selected patients with persistent exacerbations despite optimized inhaled therapy (Global Initiative for Chronic Obstructive Lung Disease, 2025).

This narrative review aims to synthesize the evidence supporting personalized pharmacological escalation in COPD. We first discuss the rationale for SITT, integrating pivotal trial evidence with current real-world evidence (RWE). We then contextualize the emergence of biologic therapy, including the pivotal mepolizumab and dupilumab trials, and discuss tozorakimab as an investigational anti–IL-33 strategy that may broaden biologic therapy beyond currently defined eosinophilic/type 2 COPD phenotypes. Finally, we frame these advances within the treatable-traits paradigm, highlighting how biomarker- and phenotype-driven strategies may help reduce the persistent gap between guideline recommendations and routine clinical practice. A summary of the key clinical trials and observational studies is provided in Table 1.

**TABLE 1:** Key evidence supporting personalized pharmacological escalation in COPD.

Evidence domain	Key studies	Population/setting	Main message	Clinical implication
Pivotal evidence for SITT	IMPACT, ETHOS	Symptomatic COPD with exacerbation history	Fixed triple therapy reduces moderate-to-severe exacerbations compared with dual inhaled regimens; mortality signals require cautious interpretation	Establishes SITT as an evidence-based inhaled escalation strategy
SITT vs. MITT in real-world care	Alcázar-navarrete; SKOPOS-MAZI	Routine-care COPD cohorts	SITT is associated with better persistence and lower exacerbation, mortality or cardiopulmonary event risk compared with MITT	Simplification of inhaled therapy may improve implementation and outcomes
Early post-exacerbation optimization	MITOS EROS + CP; ReS database	Post-exacerbation/post-hospitalisation setting	Delayed initiation and underuse of SITT remain common after severe exacerbations	Structured post-exacerbation review is needed
Approved biologics for eosinophilic/type 2 COPD	BOREAS, NOTUS, MATINEE	COPD with blood eosinophils ≥300 cells/µL and persistent exacerbations despite triple therapy (chronic bronchitis was a defining inclusion criterion for BOREAS/NOTUS, whereas MATINEE selected primarily on an eosinophilic/exacerbation phenotype, without requiring chronic bronchitis)	Dupilumab and mepolizumab reduce exacerbations in selected eosinophilic/type 2 COPD populations	Biologics are targeted add-ons, not general COPD therapy
Real-world biologic experience	Freund; lupia; mari	Small real-life dupilumab cohorts	Early data suggest feasibility and potential benefit in patients under-represented in RCTs	Registries are needed to define response criteria, safety and discontinuation strategies
Emerging non-canonical type 2 targets	FRONTIER-4; OBERON/TITANIA/MIRANDA press releases	Anti–IL-33 strategy	Tozorakimab may broaden targeted treatment beyond eosinophilic/type 2 COPD	Promising investigational pathway pending full peer-reviewed phase 3 data

Abbreviations: COPD, chronic obstructive pulmonary disease; EOS, blood eosinophil count; ICS, inhaled corticosteroid; IL, interleukin; LABA, long-acting β_2_-agonist; LAMA, long-acting muscarinic antagonist; MITT, multiple-inhaler triple therapy; RCT, randomised controlled trial; ReS, ricerca e salute database; RWE, real-world evidence; SITT, single-inhaler triple therapy; T2, type 2.

## 2 Recent GOLD updates: triple therapy and biologics in COPD

Current GOLD recommendations emphasize exacerbation history as one of the strongest determinants of future risk of exacerbation and progressively lower the threshold for optimizing treatment. In particular, a single moderate exacerbation, even when managed in the outpatient setting, is now sufficient to identify a patient at increased risk, whereas previous escalation algorithms were largely centered on patients experiencing at least two moderate exacerbations or one severe exacerbation requiring hospitalization (Global Initiative for Chronic Obstructive Lung Disease, 2025). Epidemiological data confirm that patients with frequent exacerbations exhibit significantly more rapid forced expiratory volume in 1 s (FEV1) decline compared with infrequent exacerbators, and that both the number and severity of exacerbations are independently associated with increased cardiopulmonary mortality (Gladysheva et al., 2010; Soler-Cataluña et al., 2005).

Over recent years, the role of ICS/LABA dual therapy in COPD has progressively narrowed, while triple therapy with ICS, LABA and LAMA has become the preferred option for patients with exacerbations in whom an ICS-containing regimen is appropriate, particularly when blood eosinophil counts suggest a higher likelihood of corticosteroid responsiveness (Global Initiative for Chronic Obstructive Lung Disease, 2025). Nonetheless, ICS use is not without risk, and has been consistently associated with an increased risk of pneumonia and other respiratory infections, a consideration that must be weighed against the exacerbation benefit when selecting patients for ICS-containing regimens (Global Initiative for Chronic Obstructive Lung Disease, 2025). In this context, SITT is generally preferred to multiple-inhaler triple therapy (MITT), as simplifying the treatment into a single device can improve adherence, persistence and inhalation technique, as well as promoting synergy between molecules. Consistently, in a 12-month comparative real-world study, SITT was associated with a 37% higher treatment persistence, a 32% lower risk of exacerbations, and a 33% lower risk of all-cause mortality compared with MITT (Alcázar-Navarrete et al., 2022). A further major development is the introduction of biologic therapies as add-on options for selected patients with COPD who continue to exacerbate despite optimized triple inhaled therapy. Mepolizumab, targeting interleukin (IL) 5, and dupilumab, blocking IL-4/IL-13 signaling through the IL-4 receptor α subunit, have shown efficacy in patients with an eosinophilic/type 2 inflammatory profile, with dupilumab evidence specifically generated in patients with chronic bronchitis. This represents a paradigm shift towards phenotype-driven pharmacotherapy in COPD, in which escalation beyond inhaled therapy is guided by exacerbation burden, inflammatory biomarkers and clinical characteristics (Global Initiative for Chronic Obstructive Lung Disease, 2025).

## 3 Real-world evidence on single-inhaler triple therapy

SITT has emerged as the preferred pharmacological strategy for COPD patients requiring ICS, supported by evidence from pivotal randomized controlled trials (RCTs) and by a rapidly expanding body of real-world evidence. Large phase 3 trials, including IMPACT and ETHOS, established the efficacy of fixed triple combinations in reducing moderate-to-severe exacerbations compared with dual inhaled regimens, with additional benefits on lung function and health status (Lipson et al., 2018; Rabe et al., 2020). Mortality signals have also been reported, although their interpretation requires caution because of trial duration and the potential influence of previous ICS use or withdrawal (Lipson et al., 2018; Rabe et al., 2020). The rationale for favoring SITT over MITT rests on three complementary pillars: greater treatment adherence, lower risk of inhaler technique errors, and an evolving evidence base showing associations with improved clinical outcomes, including exacerbation reduction and, in some studies, lower mortality and cardiopulmonary event rates (Alcázar-Navarrete et al., 2022; Pollack et al., 2025b). An Italian expert opinion paper published in 2026 synthesized evidence from both RCTs and real-world studies, concluding that fixed-dose SITT provides clinically meaningful advantages over MITT and that the current therapeutic gap in Italy, where SITT remains underused among eligible COPD patients, represents an urgent unmet need (Bianco et al., 2026).

One of the most comprehensive real-world sources of evidence for the impact of SITT on hard clinical outcomes comes from the SKOPOS-MAZI study, which compared budesonide/glycopyrronium/formoterol (BUD/GLY/FORM) Aerosphere as SITT against MITT in a large propensity-matched cohort of COPD patients (SITT n = 9,974; MITT n = 8,728) (Pollack et al., 2025b). BUD/GLY/FORM was associated with an 18% lower risk of all-cause mortality compared with MITT (weighted HR 0.82; 95% CI 0.75–0.91; p < 0.001; absolute risk reduction 1.5%; NNT = 68). The benefit was consistent across all prespecified subgroup analyses. Beyond survival, BUD/GLY/FORM was associated with a 12% lower risk of severe cardiopulmonary events versus MITT (HR 0.88; 95% CI 0.83–0.93; ARR 2.1%), a finding of particular clinical relevance given the well-established bidirectional relationship between COPD exacerbations and cardiovascular morbidity (Pollack et al., 2025b). However, no head-to-head RCT has been conducted comparing MITT and BUD/GLY/FORM Aerosphere; therefore, the observational nature of SKOPOS-MAZI necessitates caution in causal interpretation.

Consistent findings emerge from the Spanish real-world ORESTES study, a retrospective multicenter observational study of 718 patients with COPD initiating BUD/GLY/FORM in routine clinical practice (Alcázar-Navarrete et al., 2025). The cohort was characterized by advanced disease burden: 72.3% of patients had a high-risk phenotype, 78.4% reported significant dyspnea (mMRC ≥2), 93.0% had ≥3 comorbidities, and 79.7% had at least one cardiovascular comorbidity. Despite this high complexity, 89.3% of patients completed 12 months of BUD/GLY/FORM treatment. After initiation, moderate and severe exacerbation rates decreased by 20.8% and 23.1% respectively, rescue medication use declined by 21.3%, and hospitalizations decreased by 24.7%. A subsequent sub-analysis specifically evaluated patients stepping up from dual therapy or switching from previous triple therapy to BUD/GLY/FORM, reporting annualized exacerbation rate reductions of 11.6% and 15.5%, respectively, with reductions of 17.8% and 14.1% among patients previously receiving SITT and MITT, respectively (Figueira-Gonçalves et al., 2026). These findings are consistent with clinical benefit of BUD/GLY/FORM across a broad spectrum of treatment pathways in real-world practice, although, as an observational sub-analysis, they do not establish a causal effect.

A head-to-head real-world comparison of two commonly used SITT formulations—fluticasone furoate/umeclidinium/vilanterol (FF/UMEC/VI) and BUD/GLY/FORM—was provided by a large retrospective study using United States Medicare claims data (FF/UMEC/VI n = 10,093; BUD/GLY/FORM n = 3,926) in patients stepping up from dual bronchodilator therapy (Wedzicha et al., 2025). FF/UMEC/VI was associated with an 18% significantly lower annualized rate of moderate-to-severe exacerbations compared with BUD/GLY/FORM (0.80 vs. 0.98 events per patient-year; rate ratio 0.82; 95% CI 0.77–0.88; p < 0.001) and with a 14% lower risk in time-to-event analysis (HR 0.86; 95% CI 0.81–0.92; p < 0.001). All-cause mortality at 12 months was also 18% lower with FF/UMEC/VI (HR 0.82; 95% CI 0.68–0.99; p = 0.040). These data raise the possibility that differences between available SITT formulations may influence clinical outcomes in selected populations; however, these findings should be interpreted as hypothesis-generating, given the retrospective claims-based design and the potential for residual confounding.

The timing of SITT initiation after an exacerbation is an additional and often overlooked determinant of downstream outcomes. The MITOS EROS + CP studies showed that prompt initiation of BUD/GLY/FORM following a COPD exacerbation was associated with a 26% lower annualized exacerbation rate compared with delayed initiation, and a 31% lower rate compared with very delayed initiation (Pollack et al., 2025a; Strange et al., 2023). This observation is of direct clinical relevance given the data from the Italian ReS administrative database, which documented a mean time to triple therapy initiation of 31–42 days after a severe exacerbation, with only 23% of patients having initiated SITT within 12 months of hospital discharge (Ronconi et al., 2026). The same database reported a 12-month all-cause mortality of 25.6% after severe exacerbation, with 53% of deaths occurring within the first 30 days—a pattern that underscores the sentinel nature of the hospitalization event and the critical importance of optimizing therapy at the time of or immediately after discharge (Ronconi et al., 2026). Taken together, these datasets support the integration of systematic post-exacerbation review protocols into clinical pathways, ensuring that eligible patients are transitioned to SITT without delay.

Complementary evidence for the extrafine triple formulation beclometasone dipropionate/formoterol fumarate/glycopyrronium (BDP/FF/G) comes from a systematic review and meta-analysis of seven real-world observational studies (N = 5,952 patients), which reported significant reductions in CAT score (pooled reduction approximately 6 points, exceeding the MCID of 2 points) and FEV1 improvements above 100 mL (also exceeding the MCID) (Rogliani et al., 2025). These functional gains were corroborated by a European pooled analysis of six prospective observational studies, which confirmed significant CAT score reductions at 3 and 6 months (p < 0.0001), meaningful FEV1 improvements, and exacerbation-free rates of 91.1%, 83.9%, and 64.3% at months 3, 6, and 12, respectively (Brusselle et al., 2026). The extrafine particle size of BDP/FF/G enables deposition in peripheral airways—a pathologically relevant target given the early and sustained involvement of small airways in COPD—and may contribute to the functional benefit observed across spirometric and plethysmographic parameters (Usmani et al., 2021).

Emerging evidence also suggests that the choice between available SITT formulations may be refined by the individual patient’s functional profile, although this area remains exploratory. A Japanese observational study comparing FF/UMEC/VI and BUD/GLY/FORM identified distinct predictors of bronchodilatory response: FF/UMEC/VI appeared more effective in patients with severe symptoms (higher mMRC score) and greater hyperinflation (lower % inspiratory capacity), while BUD/GLY/FORM appeared more effective in mildly symptomatic patients with better-preserved inspiratory capacity (Ohtsuka et al., 2023). Italian real-world data reinforce the central mechanism of deflation: a real-life cohort of 44 patients with frequent exacerbations treated with FF/UMEC/VI for 24 weeks demonstrated significant reductions in residual volume alongside improvements in FEV1, FVC, mMRC, CAT, and exacerbation rate (all p < 0.001), with functional changes significantly correlated with clinical improvements (Pelaia et al., 2021). Consistent with these findings, a further Italian real-world 12-month analysis confirmed that FF/UMEC/VI was associated with disease stability, with sustained improvements in lung function and symptom control over the follow-up period (Lupia et al., 2026b). A further Italian real-world study on 110 patients treated with umeclidinium/vilanterol dual bronchodilation for 52 weeks confirmed that improvements in airway resistance and lung volumes are closely coupled with gains in symptom scores and exacerbation reduction, lending additional mechanistic support to the deflation hypothesis as the shared pathway of benefit across LABA/LAMA-containing regimens (Pelaia et al., 2023). Although data from dual bronchodilation are outside the direct scope of SITT, they provide mechanistic support for the concept that improvements in airway resistance and lung volumes may translate into symptomatic and clinical benefits across LABA/LAMA-containing regimens.

Taken together, the real-world evidence reviewed in this section consistently supports SITT as the preferred strategy for COPD patients requiring ICS, across a range of clinical settings, patient profiles, and SITT formulations. The convergence of findings from large observational studies in Europe and the United States, supported by meta-analyses and pooled analyses, provides a robust evidence base that complements and, in some respects, extends the findings of the randomized trials underlying GOLD 2026 recommendations. The persistence of a substantial therapeutic gap—most starkly illustrated by the Italian ReS data—represents an urgent public health challenge, with a modelling data projection estimating that increasing SITT adoption from current levels to 45% over 10 years could prevent approximately 40,000 deaths and save over €646 million in hospitalization costs in Italy alone; these figures represent model-based projections rather than observed or demonstrated effects (Santus et al., 2025).

## 4 Biologic therapies in COPD: from rationale to clinical evidence

### 4.1 Rationale for biologic therapy in COPD

An estimated 20%–40% of patients with COPD exhibit signs of eosinophilic type 2 inflammation, characterized by elevated blood eosinophil count (Singh et al., 2022). Type 2 inflammation in COPD is sustained by partially overlapping inflammatory pathways involving IL-5, IL-4, IL-13, and upstream epithelial alarmins such as IL-33, which contribute to eosinophil biology, mucus hypersecretion and airway remodeling (David et al., 2021). Although blood eosinophilia in COPD carries important caveats—including significant intra-patient variability, potential discordance between blood and airway eosinophilia, and a cut-off of 300 cells/µL that falls within the upper range of normal—it remains the best available biomarker for predicting ICS responsiveness and biologic treatment benefit in clinical practice (Singh et al., 2022; Beech et al., 2024; Hastie et al., 2017; Turato et al., 2018; Tinè et al., 2019; Southworth et al., 2018; Pignatti et al., 2019). Importantly, eosinophilic COPD is biologically distinct from asthma: it is characterized by less dominant eosinophilic inflammation, a distinct epithelial type 2 gene signature, a weaker IgE/IL-4 allergic axis, and different mucus pathophysiology (Beech et al., 2024). Accordingly, the clinical development of biologics in COPD has been slower and less linear than in severe asthma, with several programmes showing mixed or negative results before the recent positive phase 3 trials.

Mepolizumab is a humanized monoclonal antibody that specifically targets IL-5 (anti-IL-5), a key cytokine driving eosinophil differentiation, maturation, and survival (David et al., 2021). The clinical development programme in COPD began with the phase 3 METREX and METREO trials in 2017, which reported an 18% and 20% reduction in exacerbation rates, respectively, with significance achieved only in the eosinophilic subpopulation of METREX (Pavord et al., 2017). These early trials provided an important proof-of-concept for IL-5 inhibition in eosinophilic COPD, but interpretation was limited by modest effect size, inconsistency between the two studies, and by trial populations that were not as strictly separated from asthma as later COPD biologic trials. In METREX and METREO, patients with a current diagnosis of asthma were excluded, and non-smokers with a history of asthma were excluded, but historical asthma in ever-smokers was not uniformly excluded. A subsequent meta-analysis of these trials refined the target population, identifying blood eosinophils ≥150 cells/µL at screening or ≥300 cells/µL in the prior year as the optimal threshold for treatment benefit (Pavord et al., 2021).

### 4.2 Pivotal randomized controlled trials

The negative phase 3 GALATHEA and TERRANOVA trials with benralizumab (anti-IL-5Rα), followed by the failure of the more selected RESOLUTE phase 3 trial to meet its primary endpoint, indicate that eosinophil depletion through IL-5 receptor α blockade has not translated into a confirmed maintenance treatment strategy for COPD, despite biological plausibility and *post hoc* signals in selected subgroups (Criner et al., 2019; Criner et al., 2020). Despite these mixed early results, the field changed substantially with the positive dupilumab programme, followed by the MATINEE trial of mepolizumab (Bhatt et al., 2023; Bhatt et al., 2024; Sciurba et al., 2025). Dupilumab became the first biologic approved for COPD, marking the first regulatory validation of targeted biologic therapy in this disease. Dupilumab is a fully human monoclonal antibody blocking the shared IL-4 receptor alpha subunit, thereby inhibiting both IL-4 and IL-13 signaling. The phase 3 BOREAS and NOTUS trials demonstrated respectively a 30% and 34% reduction in moderate or severe exacerbations compared with placebo in patients with COPD, blood eosinophils ≥300 cells/µL, and chronic bronchitis (Bhatt et al., 2023; Bhatt et al., 2024). Both trials excluded patients with a current diagnosis or history of asthma, thereby generating evidence in a more clearly defined COPD population than earlier anti-IL-5/anti-IL-5Rα studies. A prespecified subgroup analysis demonstrated that patients with fractional exhaled nitric oxide (FeNO) ≥20 ppb achieved greater exacerbation reductions (36% in BOREAS and 52% in NOTUS), suggesting that FeNO may serve as an additional biomarker to guide patient selection for dupilumab (Bhatt et al., 2023; Bhatt et al., 2024).

The phase 3 MATINEE trial provided definitive evidence for mepolizumab in COPD (Sciurba et al., 2025). In 804 patients with COPD (with no past history or current diagnosis of asthma), blood eosinophils ≥300 cells/µL, a history of exacerbations (≥2 moderate or ≥1 severe in the prior 12 months), and background triple inhaled therapy, mepolizumab 100 mg subcutaneously every 4 weeks reduced the annualized rate of moderate or severe exacerbations by 21% compared with placebo (rate ratio 0.79; 95% CI 0.66–0.94; p = 0.01). The Kaplan–Meier median time to first moderate or severe exacerbation was 419 days in the mepolizumab group versus 321 days in the placebo group (HR 0.77; p = 0.009), representing a 98-day delay in the first exacerbation. The annualized rate of exacerbations resulting in emergency department visit or hospitalization was numerically lower (rate ratio 0.65; 95% CI 0.43–0.96), although no formal statistical inference should be made for this endpoint because preceding patient-reported outcome endpoints in the hierarchical testing strategy did not reach significance. The safety profile was favorable, with adverse event rates similar to placebo. MATINEE was also the first biologic trial in COPD with follow-up extended to 104 weeks, providing robust long-term efficacy and safety data (Sciurba et al., 2025). Notably, patient-reported quality-of-life measures (CAT, SGRQ) did not reach statistical significance, underscoring that the primary benefit of mepolizumab in COPD is prevention of exacerbations rather than symptom relief.

### 4.3 Real-world evidence for dupilumab

Real-world data for dupilumab in COPD remain limited but informative. A prospective observational cohort study enrolled 23 patients with eosinophilic COPD (blood eosinophils ≥300 cells/µL) receiving dupilumab off label after BOREAS publication (Freund et al., 2025). Total exacerbation rates were reduced by 55%, including a significant reduction in severe exacerbations. Crucially, the cohort included patients who would not have met RCT inclusion criteria: seven were older than 80 years, four had FEV1 below 30% predicted, and seven were oxygen-dependent. In addition, while patients with a concurrent diagnosis of asthma or asthma-COPD overlap were excluded, the study did not explicitly exclude patients with a remote history of asthma, further differing from the pivotal dupilumab trials. These data suggest potential effectiveness in populations typically excluded from randomized trials, while underlining the complementary value of real-world evidence in characterizing biologic effectiveness.

Additional early real-life studies have provided complementary, hypothesis-generating data. In a compassionate-use study of 13 consecutive outpatients with eosinophilic COPD on maximal inhaled therapy, Lupia et al. reported rapid improvements after 4 weeks of dupilumab 300 mg every 2 weeks, including reductions in CAT and mMRC scores, improvement in pre-bronchodilator FEV1, reduction in residual volume, and decrease in blood eosinophils, FeNO and plasma fibrinogen, with no serious adverse events recorded (Lupia et al., 2026a). More recently, Mari et al. described a single-center compassionate-use case series of 12 patients with COPD treated with dupilumab, reporting a significant reduction in severe exacerbations, clinically meaningful improvement in lung function, and marked improvement in mucus-related symptoms (Mari et al., 2026). Although encouraging, these studies remain exploratory. Therefore, they should be viewed as complementary to, rather than confirmatory of, the pivotal trial evidence, while supporting the need for prospective registries to define real-world effectiveness, safety and response criteria for biologics in COPD.

### 4.4 Tozorakimab: an emerging non-canonical target

Tozorakimab is a fully human monoclonal antibody targeting IL-33, an epithelial alarmin released in response to cellular damage. Its mechanism of action is unique among biologics in development for COPD: it inhibits both the reduced form (IL-33RED), which drives type 2 inflammation via the ST2/IL-1RAP receptor complex, and the oxidized form (IL-33OX), which activates RAGE/EGFR signaling pathways leading to epithelial dysfunction, airway remodeling, and mucus plugging (England et al., 2023; Strickson et al., 2023). This dual inhibition raises the possibility of benefit in both eosinophilic and non-eosinophilic COPD phenotypes—the latter representing most patients with COPD. The phase 2a FRONTIER-4 signal-finding study evaluated tozorakimab in 135 patients with COPD, approximately 80% on background triple therapy (Singh et al., 2025). The primary endpoint was not met in the intent-to-treat population; however, in the prespecified LUNA subgroup (≥2 moderate or ≥1 severe exacerbation in the prior 12 months), tozorakimab was associated with a 59 mL improvement in pre-bronchodilator FEV1, a 36% reduction in COPDCompEx events, and a significant reduction in mucus plug scores. More recently, positive high-level results from the phase 3 LUNA programme have been announced for OBERON and TITANIA, with tozorakimab reducing the annualized rate of moderate-to-severe COPD exacerbations in both the primary former-smoker population and the overall population, including patients across blood eosinophil strata and lung-function severity stages (Clinicaltrials.gov, 2023a; Clinicaltrials. gov, 2023b). Positive high-level results have also been announced for MIRANDA (Clinicaltrials.gov, 2023c). However, because full peer-reviewed phase 3 results are not yet available, tozorakimab should currently be presented as a highly promising investigational anti–IL-33 strategy rather than as an established treatment option.

## 5 Treatable traits: a personalized framework for COPD management

The concept of treatable traits represents a paradigm shift in the management of chronic airways diseases, moving away from a syndromic, spirometry-centered approach towards a patient-centered, multidimensional assessment of clinically actionable biological, physiological, and behavioral characteristics (van Dijk et al., 2020). In COPD, this approach is particularly relevant because patients with similar degrees of airflow limitation may differ substantially in exacerbation risk, inflammatory profile, symptom burden, hyperinflation, mucus production, comorbidities, and response to treatment. The treatable-traits framework therefore provides a practical scaffold for personalized pharmacological escalation, while also reminding clinicians that drug therapy should be embedded within a broader, holistic assessment of modifiable pulmonary, extrapulmonary and behavioral factors (van Dijk et al., 2020). A treatable trait is defined as a characteristic of the individual patient that can be reliably identified, is clinically meaningful, and is amenable to a specific targeted intervention that improves relevant outcomes (van Dijk et al., 2020). In COPD, treatable traits span three domains. Pulmonary traits include eosinophilic airway inflammation, airflow limitation and hyperinflation, chronic bronchitis, emphysema, frequent exacerbations, hypoxemia, and hypercapnia. Extrapulmonary traits encompass cardiovascular comorbidities, osteoporosis, anxiety and depression, nutritional depletion and cachexia, skeletal muscle dysfunction, and obstructive sleep apnea. Behavioral and risk factor traits include active smoking, inhaler technique errors, poor medication adherence, and physical inactivity (van Dijk et al., 2020). Critically, these traits coexist in the same patient in multiple combinations, are relatively independent of one another, and each requires a distinct therapeutic response, underscoring the need for a structured multidimensional assessment rather than a single diagnostic label (van Dijk et al., 2020). This is especially important in COPD because comorbidities and behavioral traits may amplify dyspnea, worsen exercise limitation, increase the likelihood of exacerbation-like events, and reduce the apparent effectiveness of otherwise appropriate pharmacological therapy.

The practical application of the treatable traits framework in severe COPD, as described by van Dijk et al., follows a stepwise logic. In patients with frequent exacerbations, optimization of inhaled pharmacotherapy with SITT is the priority—fully concordant with the real-world evidence reviewed in Section 3 of this review (van Dijk et al., 2020). Where the eosinophilic phenotype (blood eosinophils ≥300 cells/µL) is confirmed and exacerbations persist on triple therapy, the addition of mepolizumab or dupilumab is now formally recommended by current GOLD recommendations, representing the first integration of biological therapy into a phenotype-driven pharmacological pathway for COPD (Global Initiative for Chronic Obstructive Lung Disease, 2025). For the chronic bronchitis phenotype, particularly in patients with severe airflow limitation and persistent exacerbations despite optimized inhaled therapy, add-on roflumilast may be considered (Global Initiative for Chronic Obstructive Lung Disease, 2025; van Dijk et al., 2020). However, its use in routine practice is often limited by tolerability issues, particularly gastrointestinal adverse events, reduced appetite and weight loss, which frequently lead to treatment discontinuation in real-world cohorts (Kim et al., 2017). Long-term azithromycin represents a separate exacerbation-prevention strategy, preferentially considered in former smokers (Global Initiative for Chronic Obstructive Lung Disease, 2025). In the emerging biologic era, azithromycin may therefore remain a pragmatic option for selected patients outside a clear type 2/eosinophilic profile, whereas biologics provide a more targeted strategy for biomarker-defined COPD phenotypes (Previtero et al., 2025). Conversely, the same framework should also help identify patients in whom further pharmacological escalation is unlikely to be beneficial, or in whom treatment should be reconsidered because of infectious risk, poor adherence, incorrect inhaler technique, or a non-eosinophilic phenotype with limited expected benefit from ICS or biologic therapy. Although the focus of this review is pharmacological, a treatable-traits approach should not be reduced to drug selection alone. Pulmonary rehabilitation remains a key intervention to improve exercise tolerance, dyspnea and health status, and may also reduce healthcare utilization when implemented after exacerbations (van Dijk et al., 2020). Similarly, persistent hypercapnia, severe emphysema with hyperinflation, malnutrition, skeletal muscle dysfunction and cardiovascular disease may all limit the symptomatic benefit of pharmacological treatment if left unrecognized. Their systematic identification is therefore essential not only to improve outcomes directly, but also to optimize the measurable impact of inhaled and biologic therapies. The treatable traits paradigm provides a conceptual bridge between population-level trial evidence and the individualized clinical decision-making required in daily practice. The eosinophilic phenotype exemplifies how a single biomarker can simultaneously predict ICS responsiveness, exacerbation risk, and biologic therapy benefit, functioning as an archetype for precision medicine in COPD (Singh et al., 2022; David et al., 2021; van Dijk et al., 2020). The identification of dominant hyperinflation as a physiological treatable trait provides the mechanistic rationale for LAMA-based bronchodilation and, in eligible patients, procedural lung deflation, with the degree of hyperinflation reduction emerging as the shared physiological pathway linking pharmacological and bronchoscopic interventions to clinical improvement (Pelaia et al., 2021; Pelaia et al., 2023; van Dijk et al., 2020). Similarly, chronic bronchitis and mucus hypersecretion may guide consideration of adjunctive anti-inflammatory or mucoregulatory strategies, whereas cardiovascular disease, anxiety, depression and deconditioning should be actively addressed because they may mimic, magnify or perpetuate respiratory symptoms despite adequate inhaled therapy.

Integration of the treatable traits framework into routine clinical practice has direct implications for the real-world evidence reviewed in this paper. The persistent therapeutic gap documented in Italian administrative databases—with only 23% of patients initiated on SITT within 1 year after a severe exacerbation—reflects in part the absence of systematic treatable traits assessments at the time of discharge (Ronconi et al., 2026). The underutilization of pulmonary rehabilitation despite robust guideline support, and the frequent under-recognition of extrapulmonary traits including cardiovascular disease, anxiety, depression, and nutritional depletion, represent missed opportunities in routine COPD care (van Dijk et al., 2020). The treatable traits framework, operationalized through structured post-exacerbation review protocols and multidisciplinary team assessment, offers a practical scaffold for closing these gaps. As biologic therapies become increasingly available for the eosinophilic phenotype, the treatable traits approach will be essential for guiding appropriate patient selection, ensuring that those with the most actionable type 2 inflammatory signature receive targeted therapy while avoiding inappropriate prescribing in patients without a demonstrable eosinophilic phenotype (Global Initiative for Chronic Obstructive Lung Disease, 2025; David et al., 2021; van Dijk et al., 2020). In this sense, treatable traits should be viewed as an implementation tool for personalized COPD pharmacotherapy: they help identify who should receive SITT, who may require add-on biologic therapy, and which comorbid or behavioral barriers must be addressed to maximize treatment response.

## 6 Summary

The convergence of guideline recommendations and real-world evidence presented in this review supports a clear message: in COPD patients with exacerbations who require ICS, single-inhaler triple therapy is the preferred strategy for delivering ICS-containing triple therapy and should be initiated as early as possible after an exacerbation. Rather than simply confirming trial efficacy, real-world studies highlight the practical determinants of benefit in routine care: adherence, persistence, inhaler technique, timing of initiation, and the ability to close the post-exacerbation therapeutic gap. However, observational comparisons between SITT and MITT, or between different SITT formulations, should be interpreted cautiously because of possible bias. The Italian ReS data reveal a concerning therapeutic gap: many patients remain undertreated even after severe exacerbations, despite the high short- and long-term risk associated with these events (Ronconi et al., 2026). This supports the need for structured post-exacerbation review pathways, in which pharmacological escalation is accompanied by reassessment of adherence, inhaler technique, eosinophil count, infectious risk, comorbidities, smoking status and rehabilitation needs.

The introduction of biologic therapies marks a major transition in COPD pharmacology, from inhaled escalation alone towards biomarker- and phenotype-driven add-on treatment. Dupilumab was the first biologic to obtain regulatory approval in COPD, while MATINEE subsequently provided robust phase 3 evidence for mepolizumab in eosinophilic COPD on background triple therapy (Sciurba et al., 2025). Together, these trials show that biologic benefit in COPD is achievable, but at the moment only in eosinophilic COPD. Early real-world dupilumab cohorts are useful because they explore populations under-represented in pivotal trials, such as patients with very severe obstruction, oxygen dependence, advanced age or high mucus burden (Freund et al., 2025; Lupia et al., 2026a; Mari et al., 2026). Nevertheless, these studies remain small, uncontrolled and hypothesis-generating. Prospective registries are needed to define real-world effectiveness, safety, response criteria, discontinuation strategies and the place of patients with remote asthma history or overlapping traits; the feasibility of conducting large, well-designed multicentre trials in respiratory disease within collaborative research networks, as previously demonstrated for corticosteroid dosing strategies in severe respiratory illness, supports the practicability of such initiatives in COPD biologic therapy (Salton et al., 2023). Looking beyond the currently approved biologics, tozorakimab represents a promising investigational anti–IL-33 strategy that may extend targeted treatment beyond currently defined eosinophilic/type 2 COPD phenotypes (England et al., 2023; Strickson et al., 2023). Positive high-level phase 3 results have been announced, but full peer-reviewed data are still required before its role in COPD can be defined.

This review itself has limitations that should be acknowledged. As a narrative rather than systematic review, article selection was not governed by a pre-specified protocol and may be subject to selection bias. The real-world evidence summarised here derives from heterogeneous data sources, including administrative claims, prospective cohorts, and compassionate-use case series, with differing designs, populations, and risk-of-bias profiles that limit direct comparability across studies. As with any body of observational and industry-sponsored trial literature, publication bias favouring positive findings cannot be excluded. Finally, the biologic and investigational treatment landscape in COPD is evolving rapidly, and some of the evidence discussed here, particularly for tozorakimab, is based on high-level or interim results that may be revised once full peer-reviewed data become available.

Overall, the evidence reviewed here supports a pragmatic model of personalised pharmacological escalation in COPD: SITT as the preferred inhaled strategy for patients who require ICS-containing triple therapy, followed—in patients with persistent exacerbations and eosinophilic phenotype—by targeted biological add-on treatment. Systematic assessment of comorbidities, rehabilitation needs, adherence, inhaler technique, and behavioural traits as well as the identification of new pathogenetic pathways which contribute to COPD onset, development, and progression remains essential to maximise treatment response and to identify further therapeutic targets (Screm et al., 2024; Confalonieri et al., 2023). In this sense, treatable traits provide the implementation framework linking trial evidence, real-world data and routine clinical decision-making.

## 7 Conclusion

The management of COPD is undergoing a profound transformation. Single-inhaler triple therapy has consolidated the role of optimized inhaled treatment in patients requiring ICS-containing triple therapy, while the approval of dupilumab and, more recently, mepolizumab marks the entry of COPD into the era of targeted biologic therapy for selected eosinophilic/type 2 phenotypes. The development of biologics acting beyond canonical type 2 pathways, such as IL-33 inhibition, may represent the next step, potentially expanding targeted treatment to a broader COPD population, including patients without a clear eosinophilic signature. The main challenge is now implementation: translating trial and real-world evidence into structured post-exacerbation review, appropriate biomarker-based patient selection, and a treatable-traits approach that integrates pharmacological escalation with management of comorbidities, adherence, inhaler technique and rehabilitation needs.

## Figures and Tables

**FIGURE 1:**
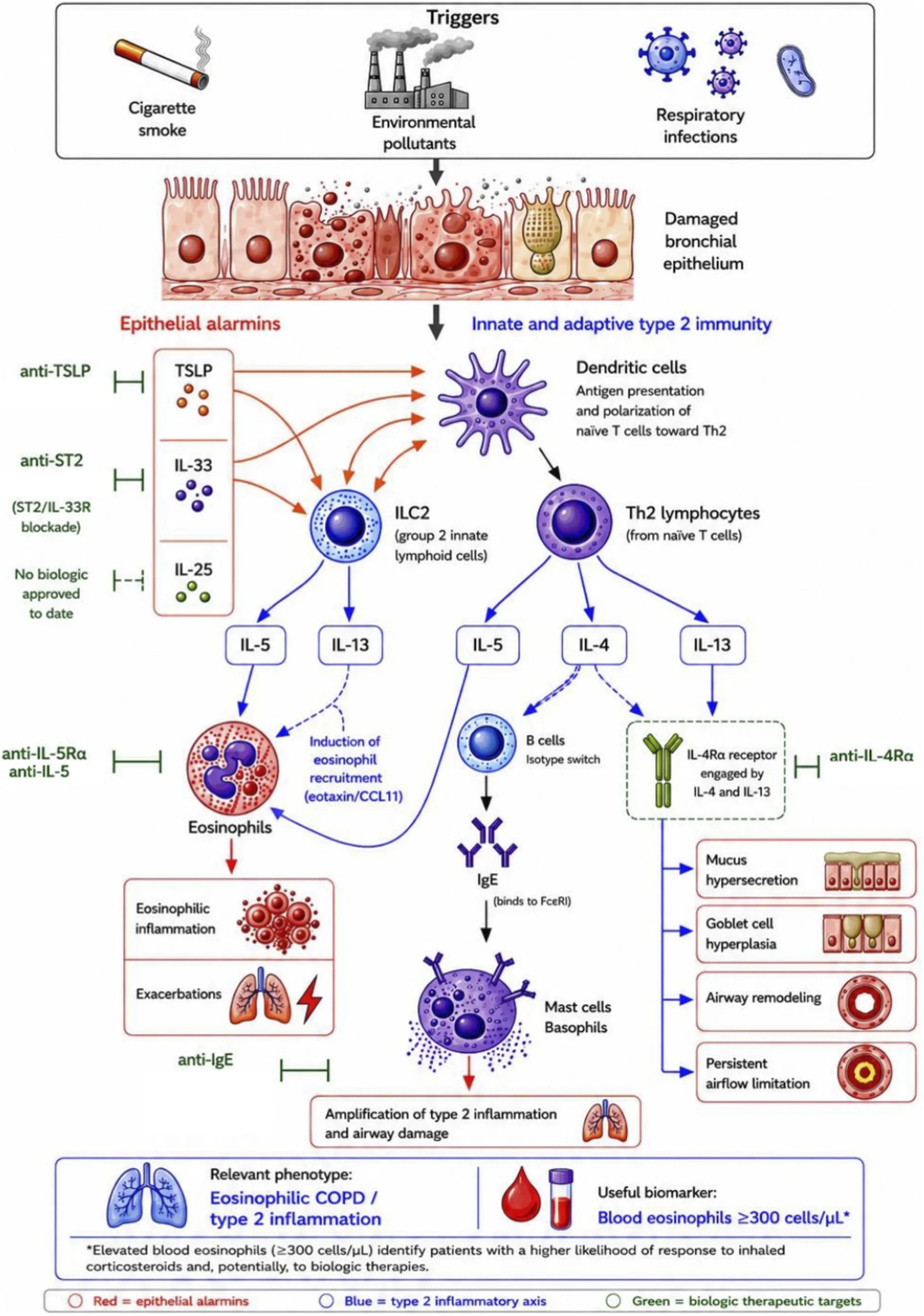
Type 2 inflammatory pathways in COPD and potential targets of biologic therapies.
